# Targeting monocyte heterogeneity in aortic aneurysms: immunomodulatory strategies and therapeutic opportunities

**DOI:** 10.3389/fcvm.2025.1670576

**Published:** 2025-09-23

**Authors:** Rasit Dinc, Nurittin Ardic

**Affiliations:** ^1^ INVAMED Medical Innovation Institute, New York, NY, United States; ^2^ Med-International UK Health Agency Ltd., Leicestershire, United Kingdom

**Keywords:** aortic aneurysm, monocyte subsets, immunotherapy, biomarker, vascular inflammation

## Abstract

Aortic aneurysms (AA) remain life-threatening vascular disorders characterized by progressive dilatation and risk of rupture. Despite advances in surgical and endovascular repair, pharmacological therapies to prevent aneurysm progression are lacking. Increasing evidence implicates chronic vascular inflammation and monocyte-derived macrophages in the pathogenesis of AA via matrix degradation, smooth muscle cell apoptosis, and neovascularization. Monocytes, traditionally classified as classical (CD14++CD16−), intermediate (CD14++CD16+), and nonclassical (CD14+CD16++) subsets, exhibit diverse functions in immune surveillance, cytokine production, and tissue remodeling. This review addresses the mechanistic roles of monocyte subsets in AA progression, evaluates emerging immunomodulatory strategies including CCR2 and TREM-1 inhibition, metabolic reprogramming, nanoparticle delivery, and cell-based therapies, and explores their integration with current surgical practices. Identification of circulating monocyte phenotypes may serve as promising biomarkers for patient stratification, monitoring, and therapeutic guidance. Advances in single-cell transcriptomics may reveal dynamic monocyte-macrophage phenotypes in aneurysm tissue. Current data hold promises for providing new perspectives on therapeutic strategies targeting monocytes. However, data are largely derived from preclinical studies. Detailed clinical studies are needed. Furthermore, translating these insights into clinical practice requires multidisciplinary collaboration among experts in immunology, vascular surgery, imaging, and systems biology.

## 1 Introduction

Aortic aneurysms (AA), including abdominal (AAA) and thoracic (TAA), are life-threatening conditions characterized by progressive aortic dilatation, wall weakening, and the risk of rupture (1). Although mostly asymptomatic, they cause approximately 150,000–200,000 deaths worldwide each year, with a mortality rate of approximately 80% in the event of aneurysm rupture (2).

Significant advances have been made in surgical and endovascular repair, but there is still no effective pharmacological treatment to prevent aneurysm progression. The current management paradigm involves surveillance until diameter-based thresholds are reached. Current practice guidelines from the Society for Vascular Surgery (SVS) in the US and the European Society for Vascular Surgery recommend elective AAA repair at a diameter of ≥5.5 cm in men (Class I-A) and at a diameter of 5.0–5.4 cm in women (2B) (3). Surgical intervention is generally recommended for TAA 5.5–6.0 cm in diameter, tailored to individual patient risk and anatomy (4). However, this diameter-based threshold leaves a significant treatment gap highlighted the need to explore underlying pathophysiological mechanisms for new, adjunctive, or alternative interventions that complement existing cardiovascular procedures.

The pathogenesis of AA involves a complex mechanism involving interactions between hemodynamic stress, genetic predisposition, and chronic inflammatory processes. Chronic vascular inflammation plays a central role in aneurysm development. Infiltration of monocyte-derived macrophages drives extracellular matrix (ECM) degradation, smooth muscle cell (SMC) apoptosis, neovascularization, and spreading inflammation, all precursors of aortic wall weakening (5, 6). Early myeloid cell infiltration in the aortic wall is considered e a hallmark of AAA development in both mice and humans, contributing to the initial steps of aortic wall destruction (7).

Recent advances in single-cell transcriptomics and systems biology have identified diverse myeloid cell populations in human AAA tissue, revealing functionally distinct monocyte/macrophage clusters, some proinflammatory and others involved in matrix remodeling (8–10). Technological advances, such as flow cytometry, have shown that monocytes consist of at least three subsets distinguished by CD14/CD16 expression: (CD14++CD16−) classical monocytes (cMo), (CD14++CD16+) intermediate monocytes (intMo), and (CD14+CD16++) nonclassical monocytes (ncMo) (11). Each subset plays distinct roles in immune surveillance, cytokine production, and tissue repair (5, 7, 12). In experimental mouse models, Ly6C^high^ monocytes, equivalent to human cMo, promote inflammatory responses and perform phagocytic functions, while Ly6C^low^ monocytes, corresponding to human ncMo, play a role in vascular patrolling, immune surveillance, and tissue repair (10). These advances have opened new avenues for precise immunomodulatory interventions.

Total circulating monocytes, particularly intMo, have been associated with aneurysm presence and progression. In a recent prospective cohort study, high levels of intermediate monocytes and monocyte-platelet aggregates predicted rapid AAA expansion over a 6-month period and improved diagnostic accuracy when combined with D-dimer levels (7). Additionally, Dinc and Ardic highlighted the lack of effective drug therapies and diagnostic biomarkers in AAA, suggesting that immunologically active subsets, such as HLA-DR^+^ monocytes, may serve as early indicators of disease progression and targets for personalized surveillance (13).

This review focuses on the emerging understanding of the role of monocyte subsets in AA pathogenesis and evaluates immunomodulatory strategies that may be alternatives or adjuncts to traditional cardiovascular interventions. In addition to targeting specific monocyte subsets for metabolic reprogramming and early aneurysm management, the role of monocyte subsets as biomarkers is also addressed.

## 2 Monocyte subsets in aneurysm pathogenesis

### 2.1 Monocyte heterogeneity in circulation and aneurysmal tissue

#### 2.1.1 Circulating monocyte subsets

Circulating monocytes consist of three main subsets with distinct transcriptomic and functional properties: classical (cMo), intermediate (intMo), and non-classical monocytes (ncMo). They are distinguished by their CD14/CD16 expression patterns, chemokine receptor profiles, and effector properties (10, 11, 14, 15).

cMo (CD14++CD16−), CCR2^high^, and CX3CR1^low^, are efficiently mobilized from the bone marrow and migrate to inflamed vascular sites (6, 16, 17). intMo (CD14++CD16+) show high HLA-DR expression, produce abundant cytokines (IL-1β, TNF-α, IL-6), and function as potent antigen-presenting cells (11, 15). ncMo (CD14+CD16++) show high CX3CR1 expression and actively control the endothelium, typically exerting anti-inflammatory and reparative effects through IL-10 secretion and endothelial support (18).

A summary of the defining markers, chemokine receptor expression, and primary functions of these subsets is provided in Table 1.

**Table 1:** Baseline characteristics of circulating human monocyte subsets.

Cell subset	Classical	Intermediate	Non-classical	Reference
Surface markers	CD14++CD16-	CD14++CD16+	CD14+CD16++	(10, 16)
Chemotaxis	CCL2/CCR2	CCL2/CCR2, CX_3_CL1/CX_3_CR1	CX_3_CL1/CX_3_CR1	(6)
Important molecules	IL-1β	IL-1β, TNF-α, HLA-DR	IL-10, TNF-α	(11)
Frequency in monocytes	85–90%	5–10%	5–10%	(16)
Functional features	Proinflammation, high phagocytic	Proinflammation, antigen presentation (for T cell), angiogenesis	Patrolling in endothelium, anti-inflammatory, tissue repair	(10, 17, 18)
Mice equivalent	Ly6C^high^ monocytes	Ly6C^int^ monocytes	Ly6C^low^ monocytes	(10)

CCL, C-C motif chemokine ligand; CCR, C-C motif chemokine receptors; CD, cluster differentiation; IL, interleukin, TNF-α, tumor necrosis factor-α.

**Table 2:** Monocyte-targeted treatment strategies in aortic aneurysms.

Target/mechanism	Therapeutic strategy	Monocyte subset(s) affected	Preclinical/Clinical Situation	Reference(s)
CCR2–CCL2 axis inhibition (e.g., SB225002, RS504393)	CCR2 antagonists or CCL2 neutralization to block monocyte recruitment	Ly6C^high^	Preclinical (mice)	(28, 36)
TREM-1 blockade (e.g., LR12 peptide)	TREM-1 antibodies or inhibitors to reduce monocyte activation	IntMo	Preclinical (mice)	(23)
Metabolic reprogramming (e.g., metformin, dimethyl fumarate)	Ketosis prevents AAA rupture via downregulation of CCR2 and MMP balance	Ly6C^high^	Preclinical (rat)	(37)
Statin therapy	Statins to reduce MMP expression and inflammatory monocyte subsets	IntMo and ncMo	Retrospective clinical studies; ongoing trials	(42, 43)
IL-1β signaling	Canakinumab (monoclonal antibody) to block IL-1β mediated inflammation	IntMo	Phase III CANTOS trial (CVD); AAA data are limited	(44)
Nanoparticle-based CCR2 targeting	Targeted delivery of RNA/siRNA to CCR2^+^ monocytes via lipid	Ly6C^high^	Preclinical (cardiac and vascular models)	(8, 38)
Cell-based therapies (e.g., M2 macrophage transfer)	Adoptive transfer of anti-inflammatory cells	Regulates vascular inflammation and supports repair	Preclinical (murine)	(41)

#### 2.1.2 Insights from single-cell transcriptomics

Single-cell RNA sequencing (scRNA-seq) has significantly advanced the understanding of monocyte heterogeneity in AAA. Recent studies have demonstrated an increase in cMo and intMo populations in both AAA blood and tissues, and an enrichment of interferon-inducible inflammatory programs (5, 8, 9). In aneurysm tissue, cMo subsets have shown upregulation of MMPs, CCL2, and S100A8/A9, linking them to extracellular matrix degradation and thrombo-inflammatory activity (5, 8, 19).

In contrast, ncMo signatures are relatively underrepresented in aneurysmal tissue, suggesting impaired recruitment or functional exhaustion during chronic inflammation (8, 18). This imbalance between inflammatory (cMo/intMo) and reparative (ncMo) subsets may underlie the predominance of destructive immune activity in progressing aneurysms (6, 18, 20).

#### 2.1.3 Monocyte-macrophage differentiation in the aneurysm wall

After incorporation into the aneurysm wall, monocytes differentiate into macrophages, whose phenotype largely reflects their subset of origin: cMo-derived macrophages acquire a proteolytic, proinflammatory phenotype characterized by the production of MMP-2, MMP-9, and reactive oxygen species (21, 22). intMo-derived macrophages further enhance adaptive immune responses by maintaining cytokine release and antigen presentation (23, 24). ncMo-derived macrophages, although less frequent, contribute to vascular repair by secreting IL-10, stimulating collagen synthesis, and promoting extracellular matrix stabilization (25, 26).

Distinguishing these lineage relationships avoids the confusion between monocytes and macrophages, and emphasizes the continuum extending from circulating subsets to tissue-resident effector populations.

#### 2.1.4 Functional implications in aneurysm biology

Monocyte heterogeneity shapes many aspects of aneurysm pathophysiology:

• **Inflammatory amplification**: cMo and intMo drive cytokine production and perpetuate chronic vascular inflammation (5, 6, 23).
• **Vascular remodeling**: Monocyte/macrophage-derived MMPs promote extracellular matrix degradation and smooth muscle cell apoptosis (21, 22, 24).
• **Thrombo-inflammatory activation**: Monocytes contribute to intraluminal thrombus propagation, fueling proteolysis and inflammation (19, 22).
• **Endothelial repair**: ncMo provide protective surveillance and IL-10-mediated stabilization, but their repair role appears to be weakened in advanced aneurysms (18, 25, 26).

**Figure 1:**
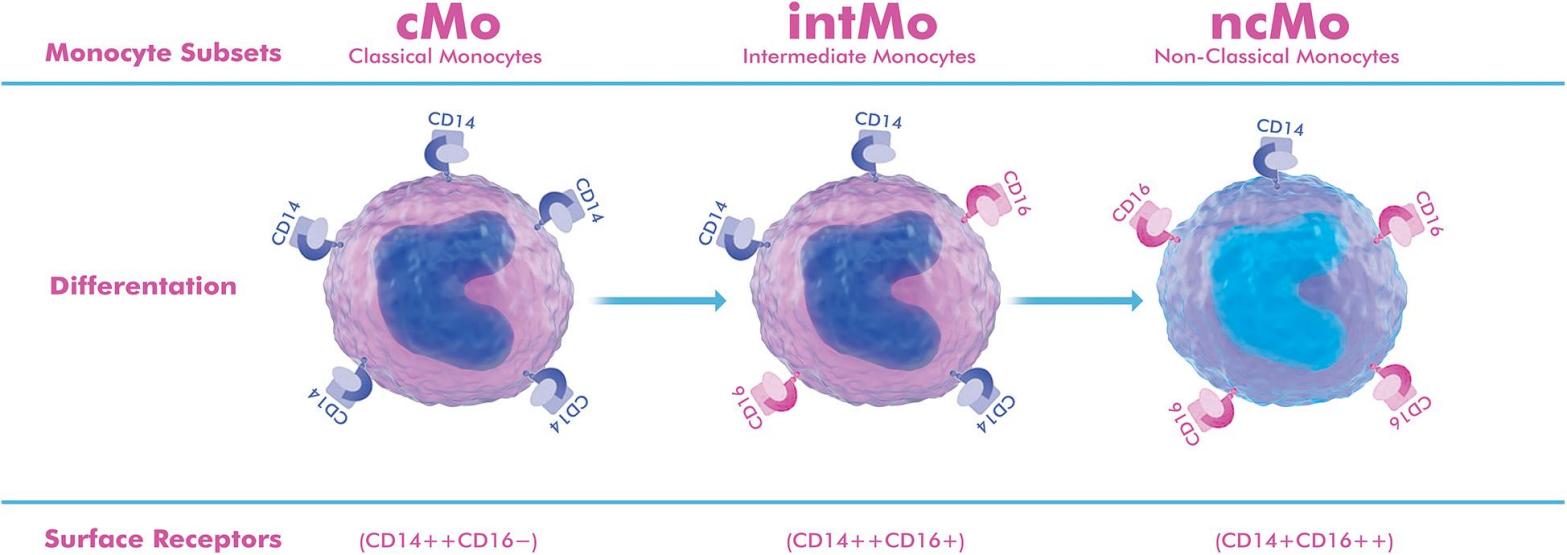
Schematic representation of monocyte subgroups based on surface markers. Classical monocytes (cMo, CD14++CD16−) constitute 85%–90% of circulating monocytes, show high CCR2 expression, and are rapidly attracted to inflamed vascular sites, where they differentiate into proinflammatory macrophages that produce matrix metalloproteinases (MMP-2, MMP-9) and cytokines. Intermediate monocytes (intMo, CD14++CD16+) constitute 5%–10% of circulating cells, show strong HLA-DR expression, and act as potent cytokine producers (IL-1β, TNF-α, IL-6), maintaining chronic inflammation and antigen presentation within the aneurysm wall. Non-classical monocytes (ncMo, CD14+CD16++) also constitute approximately 5%–10% of monocytes, show high CX3CR1 expression, patrol the endothelium, and exert reparative and anti-inflammatory functions, typically through IL-10 release and promotion of endothelial integrity. The drawing was created using the Adobe Creative Suite Package [(Illustrator, version 28.7.1 and Photoshop, version 25.12) (Adobe Systems Incorporated, San Jose, CA, USA)].

This scRNA-seq-driven framework highlights that monocyte subsets have non-redundant roles in aneurysm biology, strengthening the rationale for subset-specific therapeutic targeting. These functional roles of monocyte subsets in aneurysm biology are summarized schematically in Figure 1.

### 2.2 Infiltration of monocyte subsets into the vessel wall

The recruitment of circulating monocytes to the aneurysmal vessel wall is a tightly regulated process involving chemokines, adhesion molecules, and endothelial activation. Different subsets follow distinct signaling pathways that influence their localization and downstream functions.

#### 2.2.1 Chemokine-driven recruitment

cMo are activated from the bone marrow and spleen via CCR2–CCL2 signaling, resulting in rapid accumulation at sites of vascular injury (5, 6, 27). Endothelial adhesion molecules such as VCAM-1 and ICAM-1 further facilitate their transmigration (21, 22). intMo also respond to CCR2 but are also dependent on the CXCR4 and CCR5 pathways, which help them maintain their presence within the inflamed vascular niche (6, 23, 24). ncMo display high CX3CR1 expression and preferentially interact with endothelial CX3CL1 (fractalkine), enabling patrolling along the endothelium rather than deep tissue infiltration (18, 28, 29).

#### 2.2.2 Differentiation vs. subset-retention

After entering the aneurysm wall, monocytes differentiate into macrophages, but not all subsets behave in the same way: cMo predominantly differentiate into macrophages with proteolytic and proinflammatory properties, which trigger extracellular matrix degradation and smooth muscle cell apoptosis (21, 25). intMo can differentiate into macrophages with antigen-presenting and cytokine-enhancing functions but also appear to maintain a distinct intMo-like transcriptional profile in the tissue, suggesting a lack of complete convergence to classical macrophage pools (5, 8, 9, 23). ncMo rarely accumulates in large numbers but may directly contribute to endothelial repair or lead to the formation of macrophages with reparative, collagen-producing properties (25, 26).

This diversity suggests that monocyte subsets are not completely integrated into a single macrophage population. Instead, they retain subset-specific functional signatures that shape the inflammatory, proteolytic, and reparative balance in aneurysmal tissue. Understanding these differences is critical for therapeutic targeting, as inhibition of CCR2 signaling, blockade of TREM-1 pathways, or enhancement of ncMo reparative activity may differentially impact disease progression.

## 3 Therapeutic targeting of monocyte subsets in aortic aneurysms

### 3.1 Rationale for immunomodulatory targeting of monocytes

The current management of aortic aneurysms is limited by several key factors:

**Diameter-based thresholds**: Current management relies on size thresholds for surgery, but aneurysm diameter alone may not accurately predict rupture risk (3, 4). Small aneurysms (<5 cm), especially with high inflammatory activity, can progress or rupture, while some large aneurysms (>5.5 cm) remain stable. An autopsy series showed that 13% of ruptured AAAs were ≤5.0 cm and about 60% of aneurysms >5.0 cm did not rupture (30).
**Limited pharmacologic options**: There are no proven medications to stop or reverse AAA growth, leaving patients in a treatment gap during surveillance (31).
**Surgical risk considerations**: Many patients, especially the elderly or those with other health issues, are at high risk for surgery, limiting treatment options (32).
**Late-stage intervention bias**: Current approaches often wait until aneurysms are advanced before intervening, potentially missing changes to address underlying issues earlier (33).

**Figure 2:**
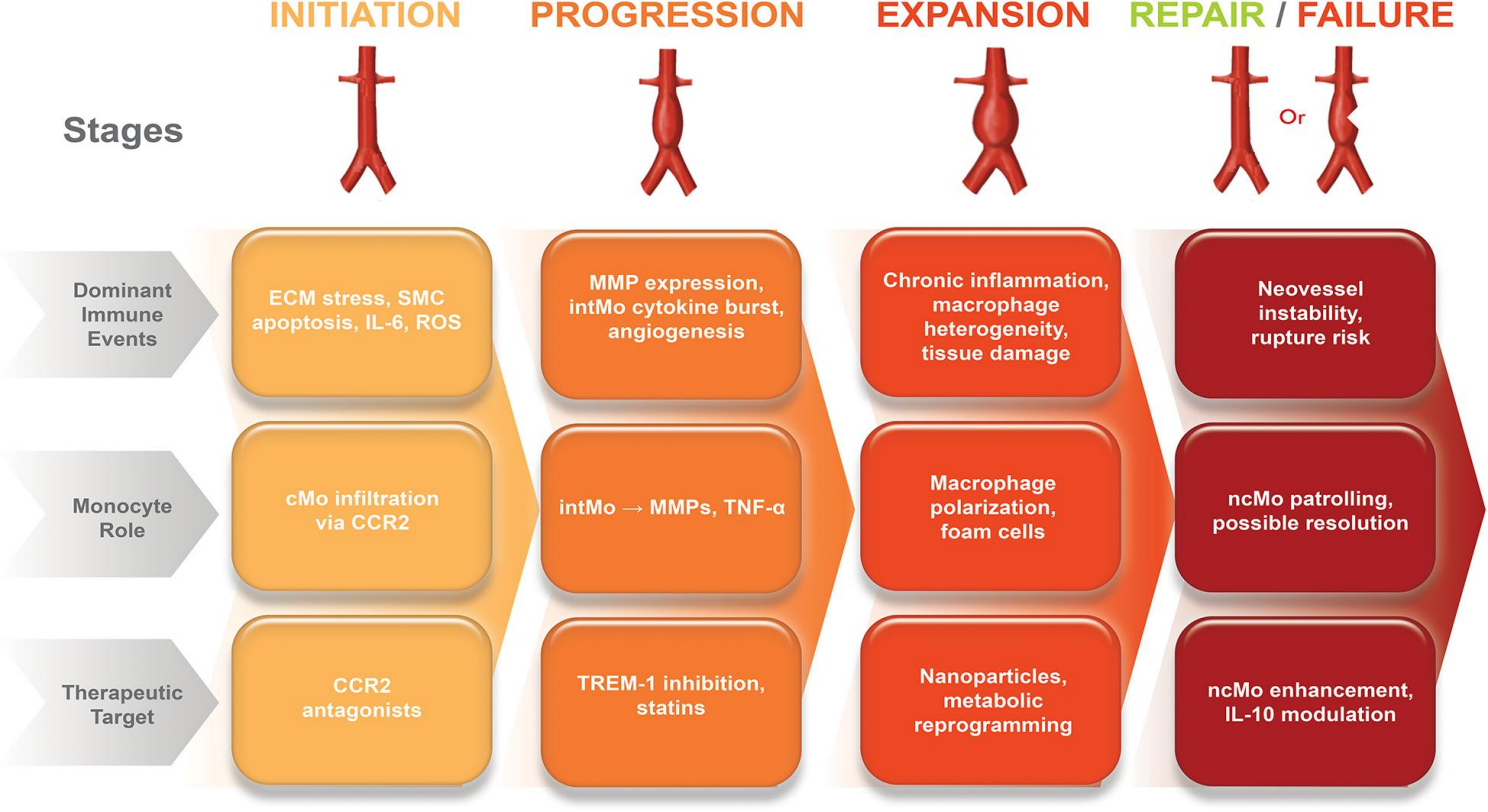
Immunological timeline of aortic aneurysm progression and monocyte-targeted therapeutic windows. The schematic illustrates the temporal sequence of aneurysm development and the predominant monocyte subsets involved. **Early stage (0–4 weeks)**: Endothelial injury activates CCR2-CCL2 signaling, leading to cMo recruitment and differentiation into inflammatory macrophages. This stage represents the optimal therapeutic window for CCR2 inhibition and TREM-1 blockade, aimed at preventing excessive infiltration and inflammatory amplification. **Intermediate stage (1–6 months)**: Persistent vascular inflammation promotes cMo accumulation and cytokine release (IL-1β, TNF-α, IL-6); strategies such as IL-1β antagonism and metabolic reprogramming are maximally effective. **Advanced stage (>6 months)**: Chronic inflammation predominates with cMo dysfunction and widespread matrix degradation; cell-based therapies, pleiotropic statins, and multimodal approaches can stabilize the aneurysm wall. This immunological timeline highlights that subset-specific interventions are most effective when matched to disease stage, with earlier targeting providing greater potential to prevent aneurysm progression. The figure was created using the Adobe Creative Suite Package [(Illustrator, version 28.7.1 and Photoshop, version 25.12) (Adobe Systems Incorporated, San Jose, CA, USA)].

In light of these gaps, there is increasing interest in a novel therapeutic strategy targeting monocyte subsets, which are key cellular mediators of aneurysm pathogenesis. Monocytes contribute to ECM degradation, SMC loss, neovascularization, and chronic inflammation through subset-specific mechanisms (5, 6, 11) (Figure 2). Therapeutic modulation of monocyte function may therefore offer a mechanistically directed, immunologically targeted adjunct to existing size-based interventions.

The goals of monocyte-targeted therapy are to: Suppress the recruitment or activation of proinflammatory cMo, reduce cytokine production from intermediate monocytes (intMo), enhance the reparative or anti-inflammatory effects of non-classical monocytes (ncMo), block key chemokine axes (e.g., CCR2/CCL2) and cytokine signaling pathways involved in monocyte trafficking and activation (23, 34, 35).

These approaches may serve as an adjunct to current management strategies. They may also delay the need for intervention in patients with early-stage aneurysms and improve outcomes in high-risk patient subgroups.

### 3.2 Emerging monocyte-targeted therapies in aortic aneurysms

As data on the phenotypic and functional heterogeneity of monocytes increases, various therapeutic approaches have emerged aiming to modulate specific subsets involved in AA pathogenesis. Current data suggest that these strategies may be a promising adjunct to surgical and endovascular care, especially for patients in the early, asymptomatic stages of the disease.

#### 3.2.1 Suppression of classical monocyte recruitment and activation

cMo, represent the largest circulating subset and are rapidly recruited to aneurysmal tissue via the CCR2–CCL2 chemokine axis. These cells differentiate into inflammatory macrophages that secrete matrix metalloproteinases (MMP-2, MMP-9), which contribute to extracellular matrix degradation and smooth muscle cell apoptosis, leading to aneurysm expansion (5, 6, 27).

Preclinical studies have consistently shown that blocking CCR2–CCL2 signaling reduces cMo infiltration into the aortic wall, reduces elastin degradation, and slows aneurysm growth. For example, CCR2 antagonists such as RS504393 and SB225002, or CCL2 neutralization, have led to significant reductions in Ly6C^hi^ monocyte recruitment and aneurysm severity in experimental models (27, 28, 35, 36).

Mechanistically, CCR2 inhibition blunts the inflammatory environment in the aneurysm wall by disrupting downstream signaling cascades involving NF-κB activation and proinflammatory cytokine release (TNF-α, IL-1β, IFN-γ) (23, 34). Importantly, CCR2 also regulates monocyte egress from the bone marrow and spleen, further linking systemic mobilization to local vascular infiltration (23, 37).

From a translational perspective, targeting CCR2^+^ subsets offer a promising strategy for early-stage aneurysms, where high inflammatory activity may be present despite subthreshold diameters (29). Furthermore, imaging with CCR2-specific PET tracers has been proposed as a diagnostic and therapeutic tool for both stratifying patients and monitoring response to therapy (33, 38).

#### 3.2.2 Reduction of intermediate monocyte cytokine production

intMo constitute approximately 5%–10% of circulating monocytes and are characterized by high HLA-DR expression and a strong capacity for antigen presentation and proinflammatory cytokine production (IL-1β, TNF-α, IL-6) (10, 11, 15). In aortic aneurysms, intMo are enriched both in the circulation and in tissue, and their persistent activation contributes to endothelial dysfunction, smooth muscle cell apoptosis, and extracellular matrix degradation, perpetuating chronic vascular inflammation (5, 6, 21, 24).

A promising strategy to mitigate this inflammatory drive is inhibition of the triggering receptor expressed on myeloid cells-1 (TREM-1), which is upregulated on activated intMo. TREM-1 signaling amplifies inflammation through immunoreceptor tyrosine-based activation motif (ITAM)-mediated pathways, resulting in NF-κB activation and increased cytokine secretion. In experimental AngII-induced AAA models, treatment with the inhibitory peptide LR12 significantly reduced Ly6Chi monocyte infiltration, downregulated adhesion molecules such as CD62l, reduced MMP expression, and ultimately attenuated aneurysm growth and elastin degradation (23).

**Figure 3:**
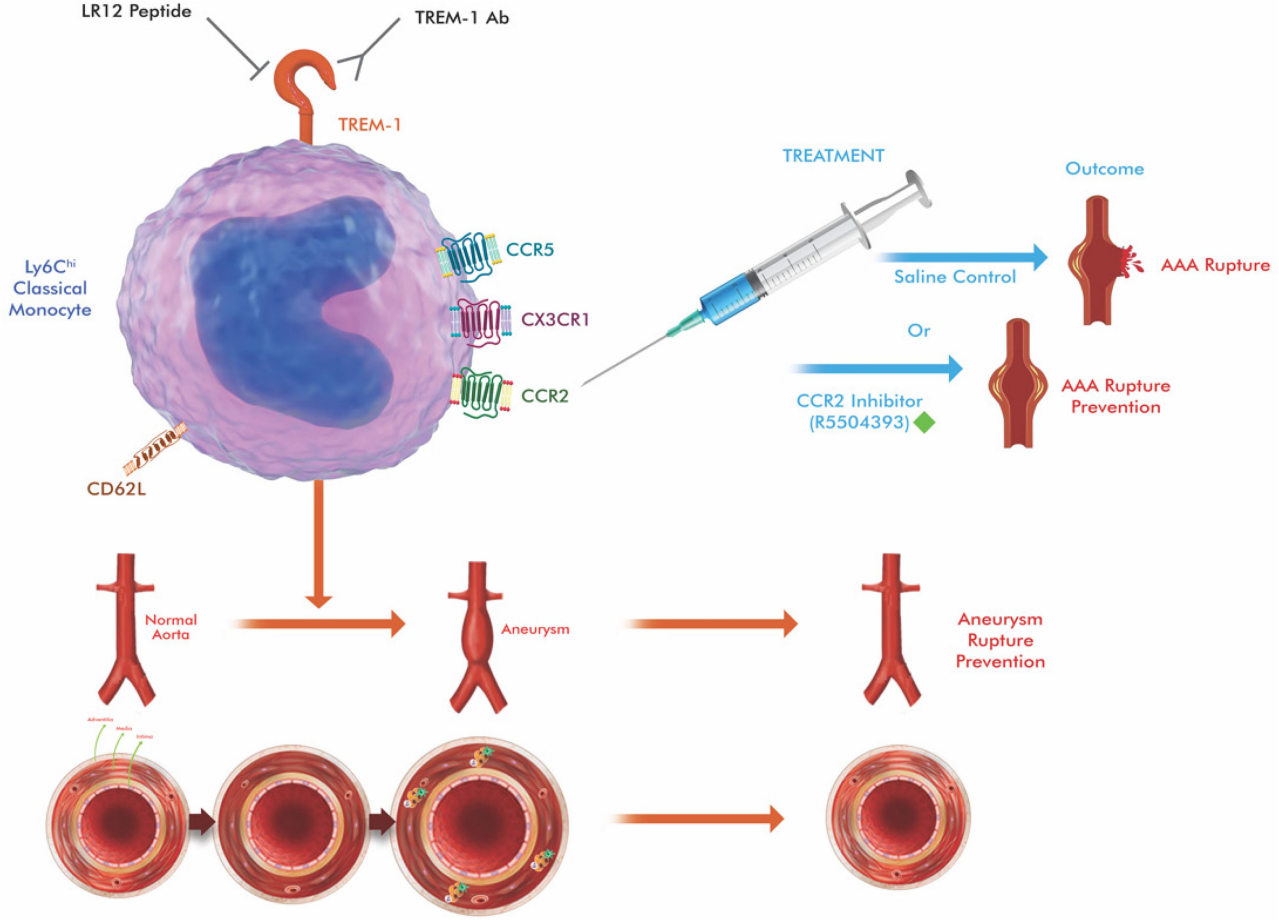
Schematic illustration of therapeutic approaches targeting at key molecules (TREM-1 and CCR2 in the figure) that regulate monocyte trafficking in aortic aneurysm. Circulating classical monocytes (cMo), which express high levels of CCR2, are normally attracted to sites of vascular injury via CCL2 gradients released by activated endothelial cells and smooth muscle cells. CCR2 antagonists (e.g., RS504393, INCB3344) block this chemokine-receptor interaction, preventing monocyte adhesion, migration, and subsequent differentiation into inflammatory macrophages. TREM-1 (Triggering receptor-1 expressed on myeloid cells) amplifies inflammatory responses in intermediate monocytes via DAP12-mediated ITAM signaling. TREM-1 inhibitors (e.g., LR12 peptide) disrupt this amplification cascade, reducing the expression of adhesion molecules (CD62l), inflammatory cytokines (TNF-α, IL-1β), and matrix metalloproteinases (MMP-2, MMP-9). The combined therapeutic approach targets both initial monocyte recruitment (CCR2 blockade) and inflammatory amplification (TREM-1 inhibition), resulting in comprehensive suppression of monocyte-mediated aneurysm progression. The drawing was created using the Adobe Creative Suite Package [(Illustrator, version 28.7.1 and Photoshop, version 25.12) (Adobe Systems Incorporated, San Jose, CA, USA)].

Therefore, modulation of intMo activity through TREM-1 blockade offers a complementary therapeutic approach to CCR2 inhibition. CCR2 antagonism primarily prevents cMo aggregation, while TREM-1 inhibition suppresses excessive cytokine release from intMo, providing a dual immunomodulatory strategy. Such combined targeting may reduce vascular inflammation, stabilize aneurysm walls, and delay disease progression, especially in patients with small aneurysms that exhibit high inflammatory activity (5, 6, 21, 23, 24) (Figure 3).

#### 3.2.3 Enhancement of non-classical monocyte repair functions

Non-classical monocytes (ncMo, CD14+ CD16++) constitute 5%–10% of circulating monocytes and are characterized by high CX3CR1 expression and vascular patrolling behavior. Under homeostatic conditions, ncMo exhibit anti-inflammatory and reparative roles, producing IL-10 and promoting endothelial repair (10, 11, 18). However, in the context of aortic aneurysms, their functions appear more complex and stage-dependent.

Clinical studies have reported increased circulating ncMo in early AAA but negative correlations with aneurysm size and volume in advanced stages (7, 27). This dichotomy suggests that while ncMo may initially contribute to vascular surveillance and stabilization, chronic inflammatory signaling may shift them toward a dysfunctional or even proinflammatory phenotype (17, 18).

Therefore, therapeutic strategies to enhance the protective and reparative potential of ncMo are being investigated. Experimental approaches include metabolic reprogramming toward anti-inflammatory phenotypes and the adoptive transfer of regulatory macrophages derived from ncMo-like populations (25, 26). Furthermore, modulating ncMo-endothelial interactions through CX3CR1–CX3CL1 signaling has been suggested to enhance vascular patrolling and reduce endothelial activation (18, 28). Harnessing the reparative functions of ncMos while preventing their maladaptive proinflammatory activation may provide a complementary therapeutic strategy to targeting CCR2 and TREM-1. This may be particularly useful in stabilizing aneurysm walls after endovascular or surgical repair, where tissue repair processes are critical (18, 39, 40).

#### 3.2.4 Metabolic reprogramming approaches

Monocyte and macrophage function is closely linked to their metabolic state. In aortic aneurysms, activated classical and intermediate monocytes often undergo a metabolic shift toward aerobic glycolysis, which promotes rapid ATP production and accelerates the production of proinflammatory cytokines and matrix-degrading enzymes (6, 20, 24). This glycolytic bias perpetuates vascular inflammation, extracellular matrix degradation, and aneurysm expansion.

Experimental studies indicate that reprogramming monocyte metabolism toward oxidative phosphorylation (OXPHOS) and fatty acid oxidation can transform these cells into an anti-inflammatory, reparative phenotype. For example, modulation of mTOR/HIF-1*α* signaling and AMPK activation has been shown to reduce IL-1β and TNF-α secretion and promote anti-inflammatory cytokines such as IL-10 (6, 20). Similarly, interventions that promote mitochondrial biogenesis and increase OXPHOS can promote the differentiation of monocytes into regulatory macrophages with pro-healing properties (25, 26).

In addition, systemic metabolic interventions such as ketogenic diets or exogenous ketone supplementation have shown efficacy in reducing monocyte-mediated inflammation and preventing AAA rupture in experimental models, in part through downregulation of CCR2 expression and improvement of extracellular matrix homeostasis (37).

While most evidence is in the preclinical stage, targeting immunometabolism offers a novel and complementary therapeutic strategy: Instead of simply blocking monocyte recruitment, metabolic reprogramming could leverage monocyte plasticity to promote vascular stabilization and tissue repair. Translational studies, including pharmacological targeting of metabolic checkpoints and dietary modulation, are needed to evaluate the feasibility of this approach in patients with aortic aneurysms (6, 20, 24, 37, 39).

#### 3.2.5 Nanoparticle-based delivery systems

Nanoparticle-based platforms have emerged as promising tools for the selective delivery of therapeutic agents to monocytes and macrophages in aortic aneurysms. Given that circulating and tissue-infiltrating monocytes actively internalize nanoparticles via phagocytosis and receptor-mediated endocytosis, these systems enable cell-specific drug targeting, enhancing efficacy while minimizing systemic toxicity (6, 20, 39).

Preclinical studies have shown that nanoparticles loaded with siRNA against CCR2 successfully silence CCR2 expression in monocytes, thereby reducing their recruitment to inflamed vascular tissue and slowing aneurysm progression (38). Similarly, nanoparticle carriers have been used to deliver anti-inflammatory agents, statins, and antioxidants directly to vascular macrophages, resulting in reduced MMP and proinflammatory cytokine production (6, 20, 39).

Nanoparticles are being investigated for diagnostic and therapeutic as well as therapeutic applications. For example, nanoparticle-based imaging probes targeting CCR2^+^ monocytes or inflammatory macrophages could improve the detection of high-risk aneurysms and potentially enable real-time monitoring of treatment response (34).

The versatility of nanoparticle systems, ranging from polymetric nanoparticles and liposomes to biomimetic vesicles, offers opportunities to customize pharmacokinetics, payload delivery, and immune cell specificity. While still largely experimental, these strategies highlight the potential of precision nanomedical approaches in the management of aortic aneurysms by reprogramming or silencing pathogenic monocyte subsets (6, 20, 34, 38, 39).

#### 3.2.6 Cell-based therapies

Cell-based approaches aim to restore vascular homeostasis and repair by exploiting the regenerative and immunomodulatory properties of immune or progenitor cells. Significant emphasis has been placed on mesenchymal stem/stromal cell (MSC) and monocyte/macrophage-derived cell therapies for aortic aneurysms.

Preclinical studies have shown that adoptive transfer of MSCs can reduce aneurysm formation by suppressing proinflammatory cytokine release, reducing MMP activity, and promoting extracellular matrix stabilization (41). MSCs exert these effects largely through paracrine secretion of anti-inflammatory mediators (e.g., IL-10, TGF-β) and modulating monocyte/macrophage polarization toward reparative phenotypes (25, 26).

Beyond MSCs, research has explored the use of ex vivo modified macrophages or monocyte-derived regulatory myeloid cells. These engineered cells can be metabolically or genetically modified to enhance their anti-inflammatory and healing-promoting properties prior to adoptive transfer, offering a way to counter the destructive immune environment in aneurysmal tissue (26, 41).

While most of the evidence remains preclinical, early data suggest that combining cell-based therapies with existing pharmacological or surgical interventions may provide synergistic vascular protection. However, challenges remain regarding cell survival, adherence, and scalability, as well as the need for long-term safety assessment in human trials (39, 41). A summary of current and emerging monocyte-targeted therapy strategies in aortic aneurysms, including their mechanisms, affected subgroups, and translational status, is presented in Table 2.

### 3.3 Integrating monocyte targeting with cardiovascular intervention

Despite advances in surgical and endovascular repair, aortic aneurysms continue to be associated with high morbidity and mortality, particularly when rupture occurs (3, 4, 32). Current practice guidelines recommend intervention primarily based on aneurysm diameter and growth rate, but these structural criteria fail to reflect the underlying biologic activity of the aneurysm wall (3, 4, 30). This limitation highlights the need for approaches that integrate monocyte-targeted immunotherapies with existing cardiovascular interventions.

#### 3.3.1 Rationale for combined therapy

Current repair strategies, open surgery or endovascular aneurysm repair (EVAR), effectively prevent rupture but do not address the persistent inflammatory and proteolytic activity that drives aneurysm growth and complications (3, 32, 40). Post-repair, residual sac inflammation and ongoing monocyte recruitment can contribute to endoleaks, graft-related inflammation, and sac expansion (40). Therefore, combining surgical repair with monocyte-targeted immunomodulation may limit postoperative inflammatory complications and promote vascular healing.

Furthermore, many patients with small aneurysms are monitored until they reach the 5.0–5.5 cm threshold, despite evidence that biologically active aneurysms can rupture below this size (30). For these patients, selective immunotherapies targeting cMo uptake, intMo cytokine release, or ncMo repair functions can effectively extend the preoperative “safe window” by delaying the risk of dilation and rupture (6, 20, 21, 23, 24, 39).

Therefore, the rationale for combined therapy aims to stabilize the aneurysm wall both structurally and immunologically, bridging the gap between biomechanical correction (surgery/EVAR) and biological modulation (monocyte-targeted interventions).

#### 3.3.2 Potential clinical applications

Several translational opportunities are emerging for the integration of monocyte-targeted therapies into cardiovascular interventions:

• **Perioperative immunomodulation**: CCR2 antagonists or TREM-1 inhibitors can be used to reduce inflammatory complications, accelerate tissue healing, and improve graft integration during EVAR or open repair (23, 25, 26, 40).
• **Adjunctive medical therapy**: Statins and anti-inflammatory agents (e.g., canakinumab) show potential to slow aneurysm progression (35, 36, 44). The addition of nanoparticle-based delivery systems, metabolic reprogramming, or cell-based therapies may enhance these benefits by specifically targeting monocyte subsets (6, 20, 34, 38, 39, 41).
• **Precise patient selection**: Molecular imaging with CCR2-specific PET tracers (32, 33) and biomarker panels (e.g., D-dimer, GDF-15, MPO) (7, 13, 24) can stratify patients based on inflammatory activity. Those with small but biologically active aneurysms may benefit from early monocyte-targeted interventions, while high-risk surgical candidates may be optimized with additional immunotherapy before or after repair.
• **Post-EVAR surveillance**: Monocyte-targeted biomarkers and imaging can complement CT or ultrasound monitoring, providing insights into biological graft responses and helping guide the need for reintervention (7, 45, 33, 40).

In general, monocyte-targeted treatment strategies are positioned as a complement, not a replacement, to conventional repair and can potentially improve outcomes across the disease spectrum.

#### 3.3.3 Immunomodulation around aneurysm repair

The acute immune activation triggered by surgical and endovascular repair of aortic aneurysms can exacerbate inflammation and sac instability. Incorporating short-term monocyte-targeted therapies (e.g., CCR2 antagonists, TREM-1 inhibitors) before and after aneurysm repair may offer benefits like suppressing macrophage-mediated matrix degradation, stabilizing the aneurysm sac post-surgery, and reducing perioperative complications such as endoleaks or sac expansion. Preoperative monocyte profiling and postoperative subset monitoring could further guide personalized immunotherapy for high-risk patients who are not suitable candidates for surgery.

## 4 Biomarkers of monocyte activation in aortic aneurysms

Biomarkers reflecting monocyte activity hold promise in improving risk stratification of AAA and TAA and complement structural imaging with biological information. Current studies focus on circulating proteins, cellular parameters, and molecular imaging approaches (Table 3).

**Table 3:** Biomarkers of monocyte activation in aortic aneurysms and their validation steps.

Biomarker	Monocyte link	Diagnostic association	Validation stage	Limitations	Reference
MCP-1/CCL2, soluble CCR2	CCR2-mediated cMo recruitment	Elevated in AAA patients; associated with dilation	Discovery	Nonspecific; overlaps with other vascular diseases	(6, 27, 35)
MMP-2, MMP-9	cMo/macrophage-derived	Predicts aneurysm growth; linked to matrix degradation	Discovery	Low specificity; affected by general inflammation	(21, 22)
GDF-15, MPO	Monocyte/macrophage activation	Higher levels in AAA; associated with disease activity	Early validation	Limited cohort size; prospective studies needed	(13, 45)
Circulating cMo and intMo counts	Expands in AAA blood	Associates with aneurysm diameter and growth rate	Preclinical validation	Technical variability in flow cytometry	(7, 12, 27)
Circulating ncMo counts	Decreases in advanced AAA	Inversely correlates with aneurysm size	Preclinical validation	Small effect size; interindividual variability	(7, 18)
CCR2-targeted PET imaging	Detects cMo uptake in tissue	Predicts aneurysm dilation in models; early human use	Early clinical translation	Expensive; not widely available	(33, 34, 38)

### 4.1 Circulating protein biomarkers

Several soluble mediators derived from monocytes and macrophages have been associated with the risk of aneurysm growth and rupture. CCL2/MCP-1 and soluble CCR2 are elevated in AAA patients and are associated with increased monocyte recruitment (6, 27, 35). MMP-9 and MMP-2, largely produced by cMo-derived macrophages, predict aneurysm expansion but lack disease specificity because they are elevated in multiple vascular pathologies (21, 22). GDF-15 and MPO have emerged as candidate plasma biomarkers with higher specificity for AAA activity (13, 45).

While most of these markers demonstrate good sensitivity in detecting aneurysm-associated inflammation, their specificity is limited due to overlap with other cardiovascular diseases. Furthermore, most are still in the discovery or early validation phase and require large, prospective cohorts before clinical application.

### 4.2 Molecular biomarkers

Flow cytometric profiling of circulating monocytes provides a more direct readout of immune activity. In AAA patients, increased frequencies of cMo and intMo are consistently reported and are associated with larger aneurysm diameter and more rapid expansion (7, 12, 27). Conversely, ncMo numbers often decrease in advanced disease and are negatively correlated with aneurysm size (7, 18).

While these cellular markers appear promising for longitudinal monitoring, technical variability in flow cytometry limits reproducibility across centers. Standardized panels and multicenter studies are needed before they can be integrated into routine practice.

### 4.3 Imaging biomarkers

Molecular imaging offers a noninvasive window into monocyte-derived inflammation. CCR2-specific PET tracers provide *in vivo* visualization of monocyte involvement and have been shown to predict aneurysm expansion in preclinical models (33, 34, 38). Hybrid PET/CT or PET/MRI protocols, by integrating structural and molecular information, could potentially guide patient selection for monocyte-targeted therapies (33, 34).

These imaging biomarkers demonstrate high specificity for monocyte-derived inflammation and are advancing toward early-stage clinical evaluation, but large-scale studies are still lacking.

### 4.4 Translational implications

Taken together, the biomarker development process progresses along a continuum:

• **Discovery phase**: protein markers (MMPs, MCP-1).
• **Preclinical validation**: flow cytometry of monocyte subsets.
• **Early clinical translation:** PET imaging with CCR2 tracers.

Future progress will depend on integrating biomarkers with clinical variables and imaging criteria to improve patient stratification. Consequently, biomarker-guided identification of biologically active aneurysms may enable earlier intervention and provide surrogate endpoints for monocyte-targeted therapy trials.

## 5 Current gaps and future directions

Despite increasing insights into monocyte heterogeneity in aortic aneurysm (AA) pathogenesis, there are several translational gaps that limit clinical application. Addressing these areas is critical for implementing monocyte-targeted diagnostic and therapeutic strategies.

Current subset classifications (cMo, intMo, ncMo) do not fully reflect the dynamic, context-dependent functional diversity of monocytes. Their phenotypes can change over time and under different inflammatory cues. Advanced single-cell transcriptomic and epigenetic approaches are needed to identify novel, therapeutically relevant monocyte states (5, 7, 46).

Most monocyte-modulating strategies remain limited to preclinical models. Rigorous early-phase clinical trials are needed to assess safety, tolerability, dosage, and biomarker-guided efficacy, especially in patients who are not candidates for surgery (6, 23).

Monocyte phenotyping should be complemented by advanced imaging (e.g., CCR2-PET/CT) and multi-omics (e.g., proteomics) to improve patient stratification. Integrated AI models can synthesize these complex datasets into clinically actionable risk scores (34, 38).

Future studies should investigate monocyte-targeted therapies in combination with standard agents (e.g., statins, antiplatelet drugs, or anti-inflammatory biologics). Synergistic effects may provide greater protection against aneurysm growth and complications (12, 39).

The advancement of this field will depend on interdisciplinary collaboration linking immunology, vascular biology, imaging, and translational research. Integrating monocyte-targeted strategies into practice will require synergy among clinicians, immunologists, bioengineers, and data scientists.

## 6 Conclusion

Recent advances have highlighted the diverse roles of monocyte subsets in the development and progression of aortic aneurysms. Monocytes play crucial role in directing matrix degradation and regulating immune responses, making them both mechanistic contributors and potential therapeutic targets. While current aneurysm management primarily relies on anatomic criteria, incorporating immunologic insights such as monocyte phenotyping and targeting could open up new possibilities for risk stratification and treatment. It is essential to continued translational efforts to validate these strategies in clinical settings and bridge the gap between experimental promise and patient outcomes. Ultimately, the translation of monocyte-targeted strategies into clinical practice will require collaborative efforts in immunology, vascular medicine, imaging, and bioengineering to enhance diagnostic methods and provide effective treatments for patients.
